# Oral Transmission of Classical Bovine Spongiform Encephalopathy in ARR/ARR Sheep

**DOI:** 10.3201/eid3112.250501

**Published:** 2025-12

**Authors:** Alvina Huor, Frederic Lantier, Jean-Yves Douet, Severine Lugan, Naima Aron, Chloe Mesic, Herve Cassard, Tomás Barrio, Hugh Simmons, Isabelle Lantier, Olivier Andreoletti

**Affiliations:** **University of Toulouse, ENVI, INRAE, IHAP, Toulouse, France** (A. Huor, J.-Y. Douet, S. Lugan, N. Aron, C. Mesic, H. Cassard, T. Barrio, O. Andreoletti); National Research Institute for Agriculture, Food and Environment, Nouzilly, France (F. Lantier, I. Lantier); Animal and Plant Health Agency, Weybridge, UK (H. Simmons)

**Keywords:** Prions, classical bovine spongiform encephalopathy, zoonoses, zoonotic risk, genetic resistance, France, United Kingdom

## Abstract

Selection for the A_136_R_154_R_171_ *PRNP* allele is known to curb classical scrapie in sheep, and we expected it to minimize the risk for classical bovine spongiform encephalopathy (c-BSE) propagation. We orally challenged newborn ARR/ARR and ARQ/ARQ lambs with ovine-passaged c-BSE. Contrary to our expectations, prion disease developed in all ARR/ARR lambs after markedly longer incubation times (≈50 months) than ARQ/ARQ controls (≈20 months). Tissue distribution of the abnormal isoform of prion protein (PrP) in clinically affected ARR/ARR sheep largely mirrored tissue distribution seen in ARQ/ARQ animals. Bioassays in bovine- and human-PrP transgenic mice showed that passage through ARR/ARR sheep did not increase the agent’s zoonotic potential. Transmission efficiency in human normal cellular isoform PrP-expressing mice remained similar to cattle c-BSE and lower than ARQ-passaged c-BSE. Our data reveal the limitations of breeding exclusively for ARR when the objective is to mitigate c-BSE risk and underscore the need to maintain specific-risk-material removal and surveillance programs.

Prion diseases, or transmissible spongiform encephalopathies (TSE), are fatal neurodegenerative disorders that occur naturally in various mammalian species, including sheep (scrapie), cervids (chronic wasting disease), and humans (Creutzfeldt-Jakob disease [CJD]). A key event in the pathogenesis of TSEs is the conversion of the normal cellular prion protein (PrP^C^), encoded by the *PRNP* gene, into an abnormal disease-associated isoform (PrP^Sc^) within the tissues of those affected. PrP^C^ is completely degraded after controlled digestion with proteinase K (PK) under nondenaturing conditions, whereas PrP^Sc^ is N terminally truncated under such conditions, leaving a PK-resistant core termed PrP^res^ (*1*).

In 1985, classical bovine spongiform encephalopathy (c-BSE), a new prion disease affecting cattle, was identified in the United Kingdom (*2*). The number of c-BSE cases in cattle rapidly increased because of the recycling of infected carcasses into the feed chain in the form of meat and bone meal (MBM) (*3*). Over the next 2 decades, c-BSE disseminated to >28 countries, mostly in Europe but also in the United States, Canada, and Japan, through the export of infected live animals and contaminated MBM and livestock feed.

Experimental oral or parenteral exposure to c-BSE demonstrated its transmissibility to sheep (*4*). Because MBM was also distributed to small ruminants, the potential spread of c-BSE in the sheep population became a major concern for health authorities. The emergence of variant CJD (vCJD) in humans, because of dietary exposure to the c-BSE agent, further reinforced those concerns, making the prevention of any potential spread of c-BSE to small ruminants a top priority in Europe (*5*,*6*).

In sheep, susceptibility to prion diseases is determined principally by polymorphisms in the *PRNP* gene. The major polymorphic sites influencing susceptibility to classical scrapie are located at codons 136 (A or V), 154 (R or H), and 171 (R, Q, or H) (*7*,*8*), which also strongly influence susceptibility to BSE. Sheep with the AHQ/AHQ and ARQ/ARQ PrP genotypes are highly susceptible to c-BSE infection when exposed through intracerebral or oral routes (*4*).

In contrast, intracerebral inoculation of ARR/ARR sheep with cattle c-BSE resulted in an inefficient transmission of the disease (incomplete attack rate), and oral inoculation failed to transmit disease or cause detectable accumulation of prion infectivity or abnormal PrP in the peripheral tissues or central nervous system (*9*). Those findings led to the conclusion that the ARR/ARR PrP genotype confers strong, if not complete, resistance to c-BSE infection in sheep. Selection for the ARR allele was originally conceived as a tool to control classical scrapie in farmed sheep population, but it also appeared to protect against possible c-BSE transmission (*10*). In this study, we experimentally exposed ARQ/ARQ and ARR/ARR newborn lambs orally to c-BSE passaged in ARQ/ARQ sheep to determine transmission efficiency of the disease.

## Materials and Methods

### Ethics Statement

All animal experiments were performed in compliance with institutional and French national guidelines (directive no. 2010/63/EU). Sheep BSE experimental transmission was approved by the local National Research Institute for Agriculture, Food and Environment committees, and mouse experiments (national registration no. 01734.01) were approved by the Ecole Nationale Veterinaire Toulouse ethics committees.

### Lamb Inoculation

We sourced sheep from New Zealand that were considered free of classical scrapie (*11*). ARQ/ARQ and ARR/ARR ewes were produced under TSE-free conditions in the United Kingdom. They were mated with ARQ/ARQ and ARR/ARR rams and exported to France. Lambs were born and raised within an A3 biosecure unit. We sequenced the *PRNP* gene of each sheep and lamb (*12*). We prepared the c-BSE inoculum by using the brainstem of 3 ARQ/ARQ sheep (at the clinical stage of the disease) that were inoculated through the intracerebral route with cattle BSE.

Lambs received 2 doses of inoculum (each equivalent to 2.5 g of brain tissue) through natural suckling. The first inoculation was received within the first 24 hours of life, and the second dose was delivered 14 days after birth. Lambs and ewes of both genotype groups were housed in a single pen. A total of 6 ARQ/ARQ and 8 ARR/ARR lambs were included in the experiment.

### Protein Misfolding Cyclic Amplification and Seeding Activity Titration

We used brain tissue from transgenic mice expressing the ovine ARQ PrP variant (tgShXI) (*13*) to prepare the protein misfolding cyclic amplification (PMCA) substrates, as previously described (*14*). We performed PMCA amplification as previously described (*14*). We included 1 to 2 unseeded controls for every 8 seeded reactions in each run. Each PMCA run included a reference ovine BSE sample (1/10 dilution series of a 10% brain homogenate) as a control for amplification efficiency. We analyzed the PMCA reaction products for the presence of PK-resistant PrP by using Western blot.

For each dilution and each sample, we tested >4 replicates in 2 independent runs. For each sample, we determined the last dilution showing >50% positive replicates (presence of Western blot–detectable PrP^res^).

We established the seeding activity titer in a reference 10% (wt/vol) frontal cortex homogenate from a clinical c-BSE ARQ/ARQ sheep by endpoint titration (intracerebral route) in bovine PrP expressing (tgBov) mice (*15*). We estimated the infectious titer (median lethal dose [LD_50_]/g IC in tgBov) by using the Spearman-Kärber method (*16*).

### Western Blot Detection of Abnormal PrP

We detected PrP^res^ by using Western blot. We conducted immunodetection by using 2 different PrP-specific monoclonal antibodies: Sha31 (1 µg/mL), which recognizes the amino acid sequences YEDRYYRE (amino acids 145–152) (*17*), and 12B2 (1 µg/mL) (*18*), whose epitope corresponds to amino acid sequence WGQGG (amino acids 89–93).

### Mouse Bioassays

We performed mouse inoculations while the mice were under anesthesia. Mice displaying clinical manifestations were anesthetized with isoflurane before being euthanized by using CO_2_ inhalation. We conducted bioassays to characterize the c-BSE strain phenotype by using tgBov mice (*15*).

We characterized c-BSE isolates’ abilities to propagate in hosts expressing human PrP by using mice expressing the methionine 129 human PrP variant (tg340-tgMet), the valine 129 human PrP variant (tg361-tgVal), and their crossbred (tgMet/Val), as previously described (*19*). We observed the inoculated mice daily and assessed their neurologic status weekly. When clinically progressive TSE symptoms were evident, or at the end of the mice lifespan, we euthanized the mice. We expressed survival time as the mean number of days postinoculation (dpi) of all the mice scored positive for PrP^res^, with a corresponding SD. In cages where no clinical signs were observed, mice were euthanized at the end of their natural lifespan (600–800 days). In those cases, incubation periods reported in the table as >600 dpi corresponded to the survival time observed in >3/6 mice.

### Lesion Profiling

We established vacuolar brain lesion profiles according to methods previously described (*20*). We created each lesion profile on the basis of data obtained from 5–6 animals. 

### Infectious Titer Estimates

We intracerebrally inoculated (20 μL) a series of 1/10 dilutions of a reference 10% (wt/vol) brain stem homogenate from an ovine-BSE (ARQ/ARQ) isolate into 6 tgBov mice. We estimated the prion infectious titer by using the Spearman-Kärber method (*16*).

## Results

### BSE Transmission

We exposed 24-hour-old ARQ/ARQ and ARR/ARR lambs to a dose of 2.5 g of infected brain (derived from cattle c-BSE intracerebrally inoculated into ARQ/ARQ sheep) through natural suckling. We administered a second dose of inoculum by the same route at 14 days of age. In each inoculated animal, we collected blood samples at different time points during the incubation phase. We euthanized animals from each genotype at 4 months postinoculation (mpi) (n = 2) and 10 mpi (n = 2 ARQ/ARQ sheep and n = 3 ARR/ARR sheep).

We observed clinical signs compatible with TSE disease in the remaining c-BSE–exposed ARQ/ARQ animals after 19 mpi and ARR/ARR animals after 48 mpi. We euthanized those animals upon showing locomotor difficulties (ARQ/ARQ at 20 mpi, ARR/ARR at 50 mpi). At necropsy, we collected brain, spinal cord, and a panel of lymphoid tissues.

**Figure 1:**
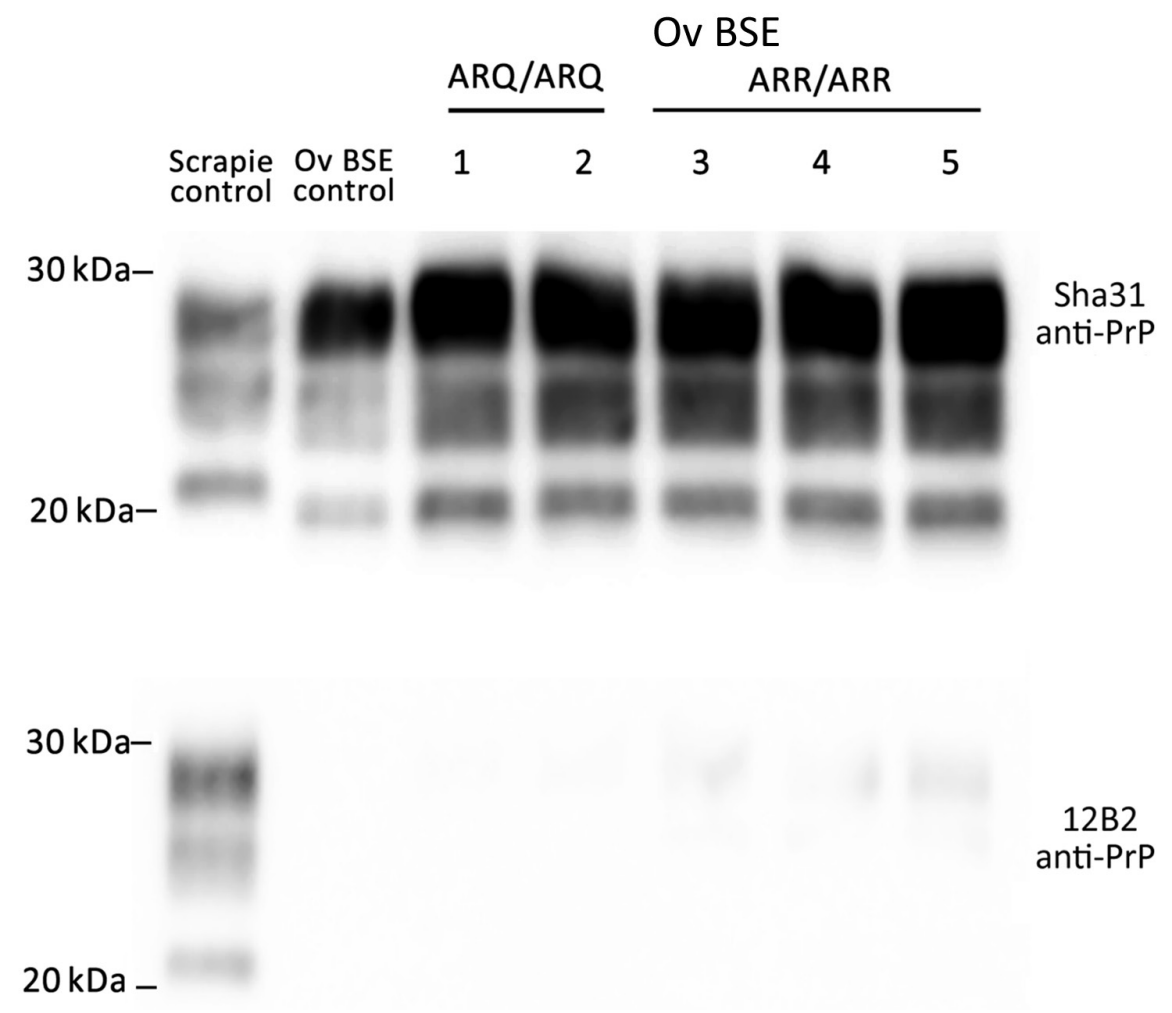
Detection of proteinase K–resistant core PrP in study of oral transmission of classical bovine spongiform encephalopathy in ARR/ARR sheep. Western blot was used for the detection of anti-PrP monoclonal antibodies Sha31 (epitope 145-YEDRYYRE-152) or 12B2 (epitope 89-WGQGG-93). BSE, bovine spongiform encephalopathy; ov, ovine; PrP, prion protein.

Irrespective of the genotype, Western blotting confirmed the presence of PrP^res^ in the posterior brainstem of each animal. The PrP^res^ Western blot banding profile displayed the typical features of the BSE agent in sheep: a 19-kDa nonglycosylated band, a dominant di-glycosylated PrP^res^ band, and an absence of immunoreactivity to the 12B2 monoclonal antibody (Figure 1).

### Ovine c-BSE PMCA Detection

PMCA is an in vitro methodology that mimics prion replication in an accelerated form, enabling amplification of minute amounts of PrP^Sc^ and prion infectivity (*21*). To determine the relative sensitivity of our optimized ovine c-BSE agent amplification PMCA protocol, we endpoint titrated a reference sample (10% cerebral cortex homogenate from an ARQ/ARQ BSE-affected sheep) by using a bioassay in tgBov mice via an intracerebral inoculation route (Appendix Table) and by PMCA by using substrates from ovine ARQ PrP-expressing mice (tgARQ/tgShXI).

The infectious prion titer of the sheep-passaged c-BSE isolate was ≈10^7.2^ LD_50_/mL IC in tgBov mice. Amplification of a 10-fold serial dilution of the same sample (12 individual replicates per dilution point) demonstrated that 2 PMCA rounds (24 h/round) were sufficient to reach the maximal sensitivity level of the assay. Additional PMCA rounds neither improved the analytical sensitivity of the assay nor increased the number of positive replicates (Appendix Figure). The estimated prion seeding titer (SA_50_) was ≈10^10.13^ SA_50_/mL by using tgARQ as substrate. Considering that mice were inoculated by using a 4-fold higher amount of material compared with the material used to seed PMCA reactions, this methodology can be considered ≈1,500-fold more sensitive than the bioassay in tgBov mice.

### c-BSE Agent Levels in Solid Tissues and Blood

**Figure 2:**
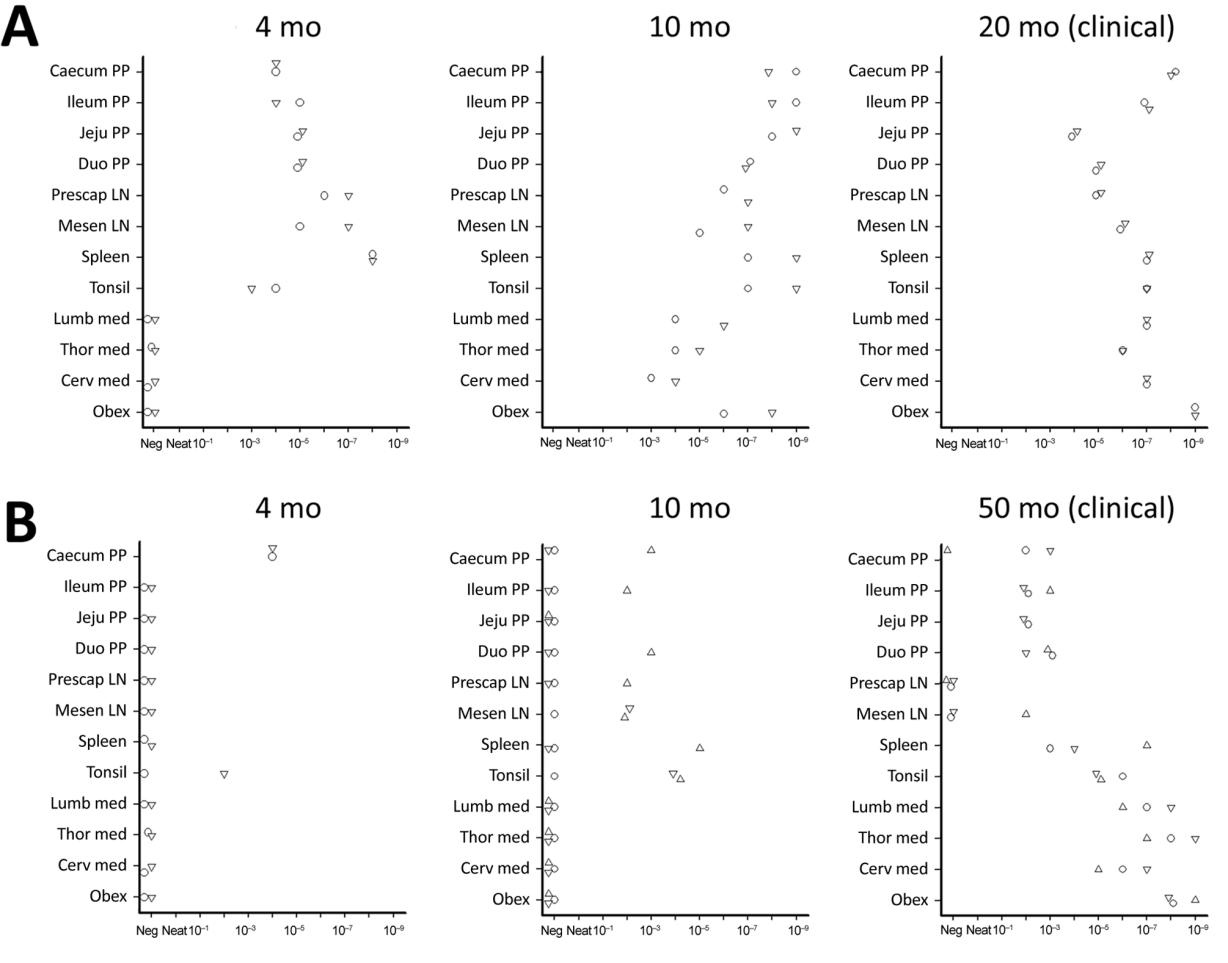
Protein misfolding cyclic amplification (PMCA) seeding activity levels in ARR/ARR and ARQ/ARQ sheep tissues after classical bovine spongiform encephalopathy challenge in study of oral transmission of classical bovine spongiform encephalopathy in ARR/ARR sheep. A) ARQ/ARQ sheep. B) ARR/ARR sheep. Protein misfolding cyclic amplification products were analyzed by using Western blot for proteinase K–resistant core prion protein detection. Each symbol represents a different animal and the associated tissue tested. Cerv, cervical; duo, duodenum; jeju, jejunum; LN, lymph node; lumb, lumbar; med, medial; mesen, mesenteric; Neg, negative; pp, Peyer’s patches; prescap, prescapular; thor, thorasic.

**Figure 3:**
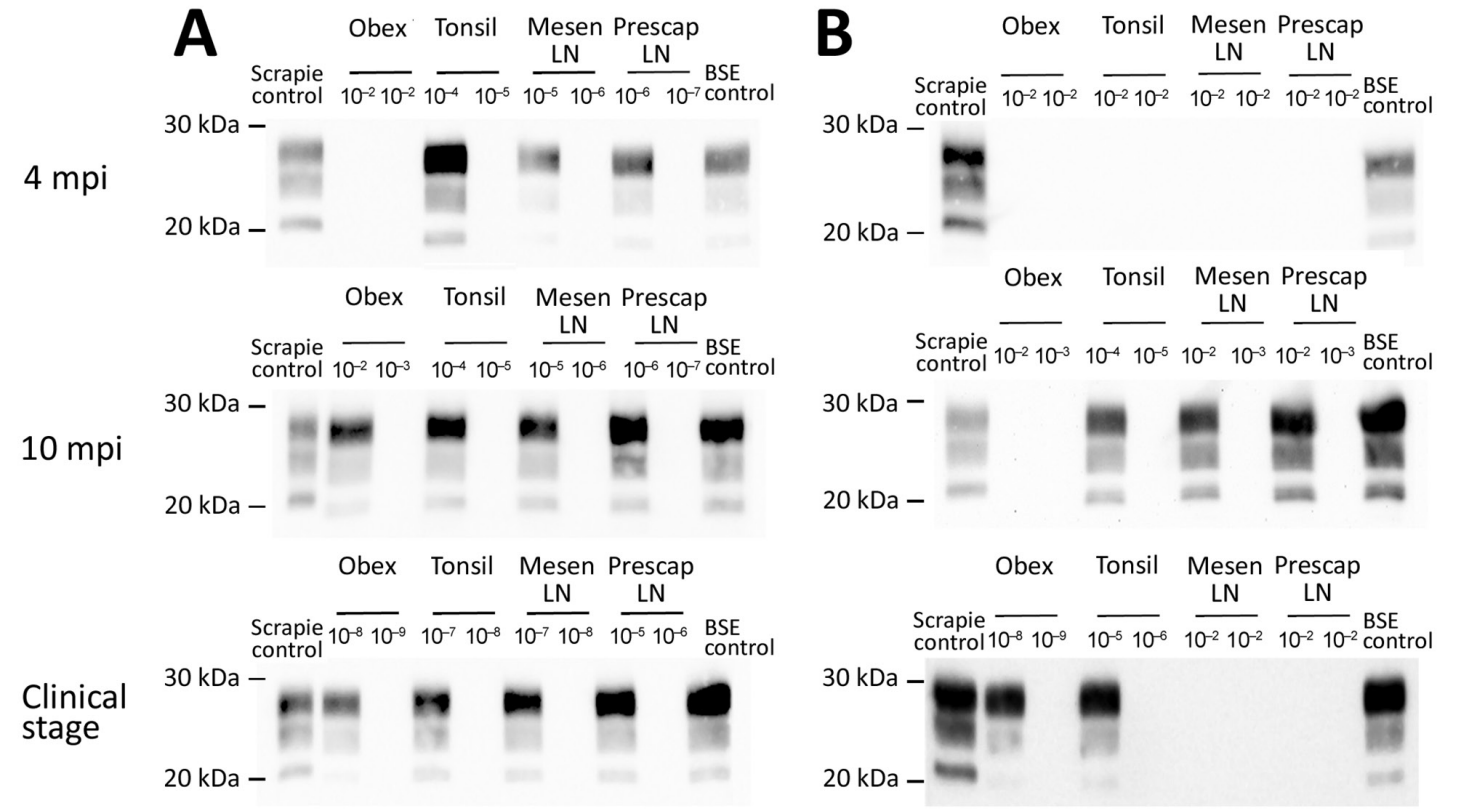
Detection of proteinase K–resistant core prion protein after 2 PMCA rounds of ARR/ARR and ARQ/ARQ sheep tissues after classical BSE challenge in study of oral transmission of BSE in ARR/ARR sheep. A) ARQ/ARQ sheep samples. B) ARR/ARR sheep samples. Western blot results from protein misfolding cyclic amplification products showing a detectable proteinase K–resistant core prion protein in >2 of 4 replicates for each tissue and animal. BSE, bovine spongiform encephalopathy; LN, lymph node; mesen, mesenteric; mpi, months postinoculation; prescap, prescapular.

**Figure 4:**
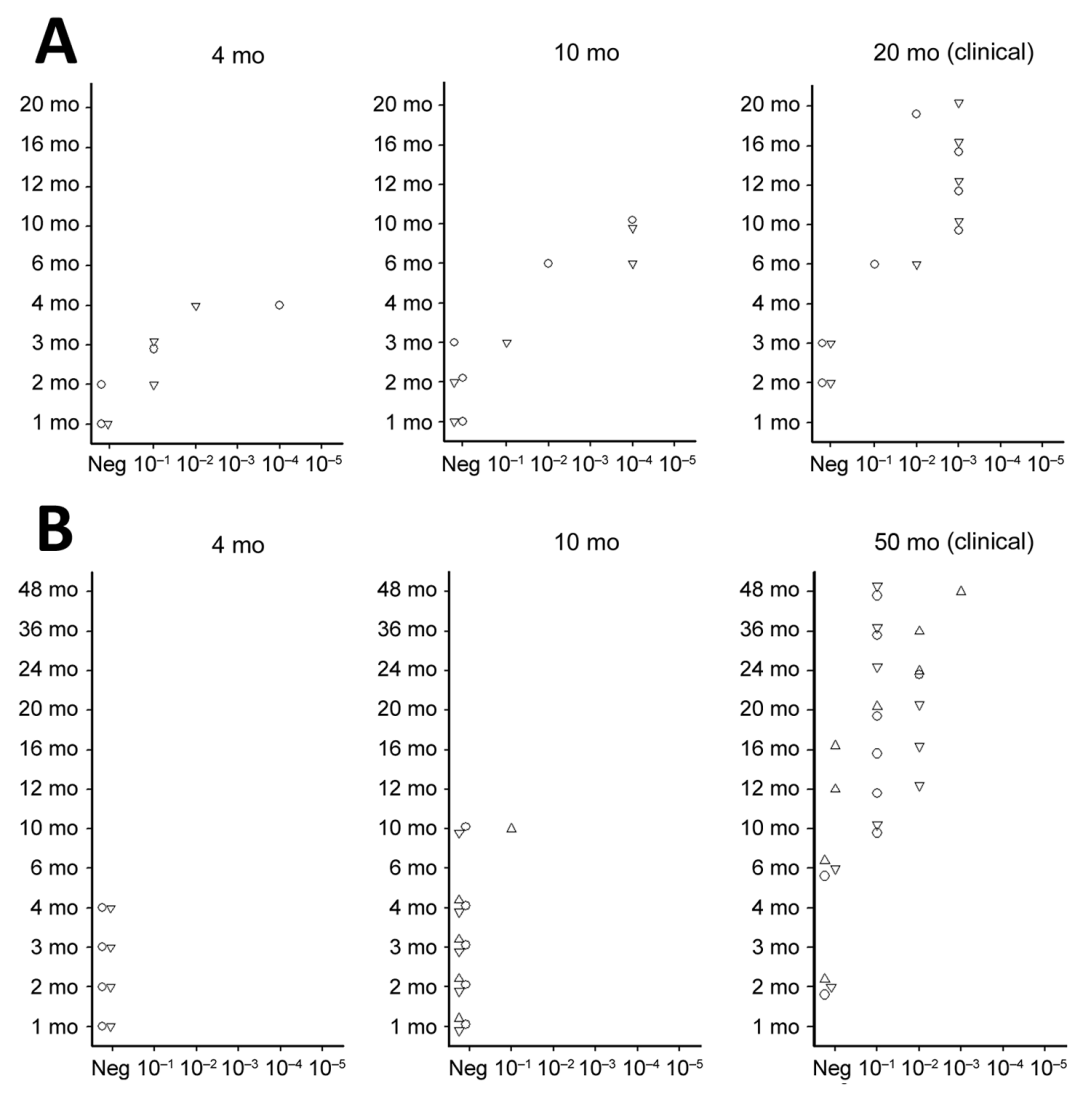
Protein misfolding cyclic amplification seeding activity levels in leukocytes of orally challenged ARR/ARR and ARQ/ARQ sheep in study of oral transmission of classical bovine spongiform encephalopathy in ARR/ARR sheep. A) ARQ/ARQ sheep. B) ARR/ARR sheep. We euthanized sheep at 4 mpi, 10 mpi, or at the clinical stage of infection. Leukocytes from 5 mL of whole blood collected at each month from birth until euthanasia were used to seed quadruplicate protein misfolding cyclic amplification reactions with a 10% wt/vol serial dilution from 10^−1^ to 10^−5^. Each symbol represents a different animal. mpi, months postinoculation; neg, negative.

We used the optimized PMCA protocol to characterize the levels of prion seeding activity in the central nervous system (CNS), lymphoid tissues, and blood collected from the c-BSE orally challenged lambs. We stored leukocytes isolated from blood samples collected from all the c-BSE-challenged animals during their incubation period (5 mL original blood equivalent) as dry pellets. We prepared 10-fold dilution series from the different samples collected from the ARR/ARR and ARQ/ARQ sheep, either 10% tissue homogenates or leukocyte pellets homogenized in PMCA buffer, and subjected them to 2 rounds of PMCA (Figure 2). We tested the presence of PrP^res^ in the amplification products from each round by Western blot (Figures 3 and 4).

### ARQ/ARQ Lambs

We detected seeding activity in all the tested lymphoid organs as early as 4 mpi. In older lambs (10 mpi and clinically affected animals), prion seeding activity in the lymphoid tissues was generally 1–4 log_10_ higher than those observed in 4-month-old animals. However, in Peyer’s patches, spleen, and tonsil, the levels of seeding activity detected at 10 mpi were higher than those measured at the clinical stage of the disease. In the CNS, seeding activity was first observed in lambs euthanized at 10 mpi. The levels of seeding activity in the CNS increased by 1–2 log_10_ in clinically affected animals (20 mpi). Of note, at the clinical stage of the disease, seeding activity in spleen, tonsil, and lymph nodes was equivalent to activity levels detected in the spinal cord and ≈2 log_10_ lower than observed in the posterior brainstem. In the blood of some animals, c-BSE seeding activity was detected as early as 2 months of age, and all animals tested at 4 months or older showed detectable levels of prion seeding activity in leukocyte samples.

### ARR/ARR Lambs

We observed low but consistent levels of seeding activity in the tonsil or cecal Peyer’s patches of the 2 euthanized 4 mpi animals. At 10 mpi, we detected seeding activity in most of the tested lymphoid organs in 2 of 3 lambs. At those stages, we detected no seeding activity in the tested CNS samples. At the clinical stage of the disease (50 mpi), we detected prion seeding activity in the posterior brainstem and spinal cord segments of the 3 tested sheep. We detected no seeding activity in the leukocytes collected at 1–10 months of age. We found positive seeding activity in 3 of 5 animals tested at 10 months of age. In animals >20 months of age, BSE seeding activity was detected in all the tested leukocyte samples.

Of note, seeding activity in the CNS of the ARR/ARR sheep was similar to those observed in affected ARQ/ARQ animals (20 mpi). At that point, we also detected seeding activity in most of the lymphoid organs. However, the levels of seeding activity were generally 1–3 log_10_ lower than those observed in the same tissues of the ARQ/ARQ affected animals.

In both ARR/ARR (10 mpi and older) and ARQ/ARQ (3 mpi and older) animals, the level of seeding activity associated with leukocyte displayed a rapid increase and then a plateau that maintained during the clinical phase. At the plateau, the levels of seeding activity in the ARR/ARR sheep leukocyte were generally lower (limit of dilution 10^−1^ to 10^−2^) than in the ARQ/ARQ sheep (limit of dilution 10^−2^ to 10^−3^).

### Strain Properties and Zoonotic Potential

**Figure 5:**
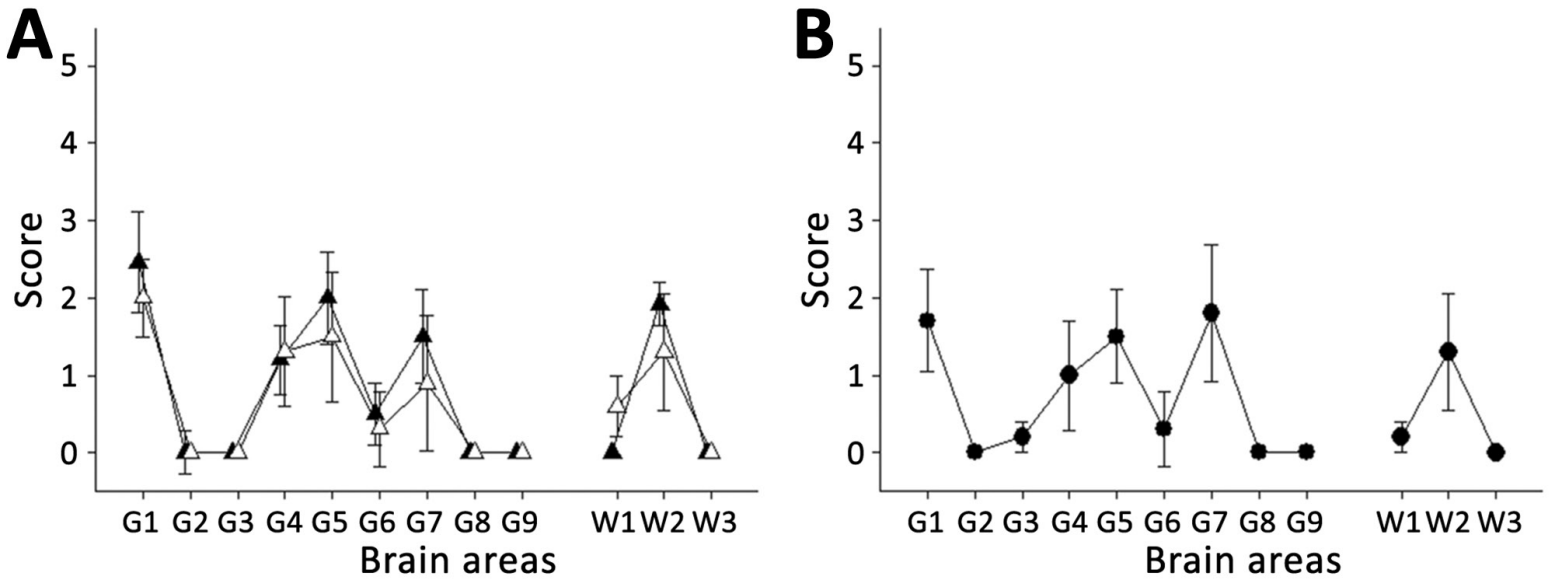
Transmission of ARR/ARR and ARQ/ARQ ovine c-BSE to tgBov mice in study of oral transmission of c-BSE in ARR/ARR sheep. A) Vacuolar lesion profiles in tgBov mice inoculated with ARQ/ARQ and ARR/ARR c-BSE isolates. B) Profiles from tgBov mice inoculated with cattle-derived c-BSE were used as controls. Lesion scoring evaluated 9 grey matter regions and 3 white matter regions. White triangles represent mice inoculated with ARQ/ARQ c-BSE isolates. Black triangles represent mice inoculated with ARR/ARR c-BSE isolates. Black circles represent mice inoculated with cattle-derived c-BSE isolates. c-BSE, classical bovine spongiform encephalopathy; G, grey matter region; tgBov, bovine prion protein–expressing mice; W, white matter region.

To characterize the potential effect of passage in ARR/ARR sheep on the strain properties of the original BSE prions, we transmitted 1 ARR/ARR and 1 ARQ/ARQ isolate (both from clinical-stage sheep, prepared as 10% posterior brainstem homogenates) to tgBov mice and performed 2 iterative passages (Table 1). On first passage, we observed some differences in survival time between the mice. However, after second passages, the survival times associated with the 2 ovine BSE isolates converged, and the vacuolar lesion profiles observed in the brains of all inoculated mice were identical to those observed in tgBov mice inoculated with cattle c-BSE isolates (Figure 5). Those results support the conclusion that passage of the c-BSE agent in ARR/ARR sheep did not alter its strain phenotype.

**Table 1:** Results of intracerebral inoculation of transgenic mice expressing bovine prion protein with a panel of bovine and ovine prion isolates in a study of oral transmission of classical bovine spongiform encephalopathy in ARR/ARR sheep*

Inoculum	Passage 1			Passage 2	
	No. positive/no. tested	Survival, dpi ±SD		No. positive/no. tested	Survival, dpi ±SD
Cattle BSE	6/6	295 ±12		6/6	265 ±35
ARQ Ov-BSE	6/6	229 ±11		6/6	237 ±5
ARR Ov-BSE	6/6	375 ±78		6/6	238 ±9
Negative brain	0/6	>750		0/6	>750
Phosphate buffered saline control	0/6	>750		0/6	>750

*BSE, bovine spongiform encephalopathy; dpi, days postinoculation; ov, ovine.

**Figure 6:**
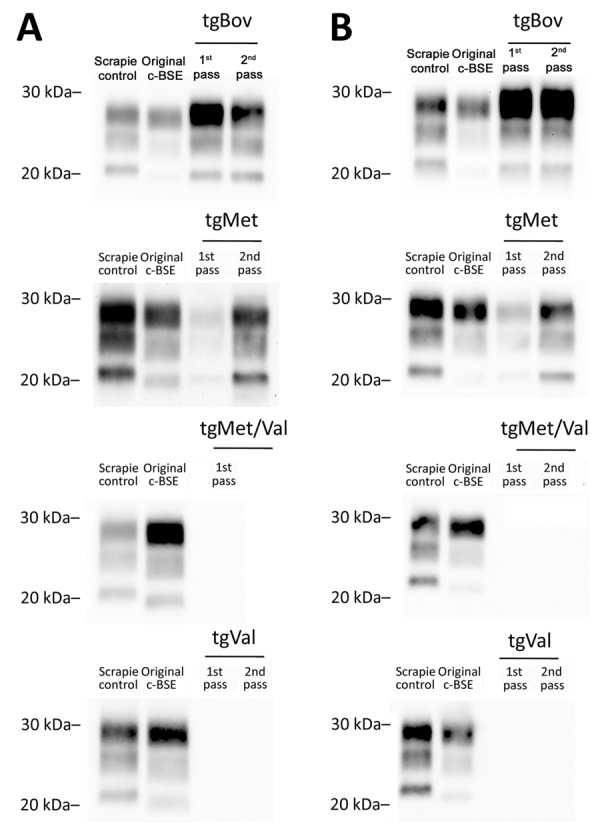
Western blot detection of proteinase K–resistant core PrP in the brains of tgBov, tgMet, (tgVal), or tgMet/Val mice inoculated with ARR/ARR or ARQ/ARQ c-BSE isolates in a study of the oral transmission of c-BSE in ARR/ARR sheep. A) ARQ/ARQ sheep. B) ARR/ARR sheep. Original c-BSE isolates from ARQ/ARQ or ARR/ARR sheep and a scrapie isolate were included as controls. Proteinase K–resistant core PrP was detected by using the monoclonal Sha31 anti-PrP. c-BSE, classical bovine spongiform encephalopathy; pass, passage; PrP, prion protein; tgBov, transgenic mice bovine PrP–expressing mice; tgMet, transgenic mice expressing human Met129; tgMet/Val, transgenic mice expressing human Met/Val129; tgVal, transgenic mice expressing human Val129.

We examined the capacity of c-BSE agents originating directly from cattle or after passage in ARQ/ARQ- and ARR/ARR-genotype sheep to replicate in humanized mice that overexpress the 3 main human PrP codon-129 variants: tgMet (Table 2), tgVal (Table 3), and tgMet/Val (Table 4). We used the same inoculum used for the tgBov experiment. We detected no transmission events (clinical disease or PrP^res^ deposition) in tgVal or tgMet/Val mice after 2 serial intracerebral passages with any of the 3 c-BSE sources. In tgMet mice, first-passage transmission was most efficient with the ARQ/ARQ-derived inoculum (4/6 mice, mean survival 560 ± 83 dpi), whereas only single, very late cases were seen with the cattle (1/6, survival >750 dpi) and ARR/ARR (1/7, survival 737 dpi) isolates. After a second passage the overall attack rate increased for all groups, but subtle kinetic differences remained: ARQ/ARQ BSE produced 100% transmission (6/6, mean survival 569 ± 55 dpi), cattle-derived BSE 50% transmission (3/6, mean survival 572 ± 64 dpi), and ARR/ARR BSE 83% transmission (5/6, mean survival 616 ± 83 dpi). Nevertheless, the PrP^res^ glycoform profile of all positive tgMet brains was indistinguishable across isolates (Figure 6), indicating that strain properties converged after adaptation.

**Table 2:** Intracerebral inoculation of tgMet humanized mice with a panel of human, bovine, and ovine prion isolates in a study of oral transmission of classical bovine spongiform encephalopathy in ARR/ARR sheep*

Isolate	Passage 1			Passage 2	
	No. positive/no. tested	Survival, dpi ±SD		No. positive/no. tested	Survival, dpi ±SD
Cattle BSE	1/6†	>750		3/6	572 ±64
ARQ Ov-BSE	4/6	560 ±83		6/6	569 ±55
ARR Ov-BSE	1/7	737		5/6	616 ±83
Negative brain	0/12	>750		0/12	>750
Phosphate buffered saline control	0/18	>800		0/12	>650

*BSE, bovine spongiform encephalopathy; dpi, days postinoculation; ov, ovine. †Abnormal prion protein isoform positive brain in a found dead animal without clinical manifestations of transmissible spongiform encephalopathies.

**Table 3:** Intracerebral inoculation of tgVal humanized mice with a panel of human, bovine, and ovine prion isolates in a study of oral transmission of classical bovine spongiform encephalopathy in ARR/ARR sheep*

Isolate	Passage 1			Passage 2	
	No. positive/no. tested	Survival, dpi ±SD		No. positive/no. tested	Survival, dpi ±SD
Cattle BSE	0/6	>750		0/6	>750
ARQ Ov-BSE	0/6	>750		0/6	>750
ARR Ov-BSE	0/6	>750		0/6	>750
Negative brain	0/12	>750		0/6	>750
Phosphate buffered saline control	0/12	>750		0/6	>750

*BSE, bovine spongiform encephalopathy; dpi, days postinoculation; ov, ovine.

**Table 4:** Intracerebral inoculation of tgMet/tgVal humanized mice with a panel of human, bovine, and ovine prion isolates in a study of oral transmission of classical bovine spongiform encephalopathy in ARR/ARR sheep*

Isolate	Passage 1			Passage 2	
	No. positive/no. tested	Survival, dpi ±SD		No. positive/no. tested	Survival, dpi ±SD
Cattle BSE	0/6	>750		NA	NA
ARQ Ov-BSE	0/6	>750		NA	NA
ARR Ov-BSE	0/6	>750		0/6	>750
Negative brain	0/12	>750		0/6	>650
Phosphate buffered saline control	0/12	>750		0/6	>650

*BSE, bovine spongiform encephalopathy; dpi, days postinoculation; NA, not available; ov, ovine.

Our observations demonstrate that passage through ARR/ARR sheep does not abolish the zoonotic capacity of c-BSE but appears to impose a modest additional barrier. That barrier is manifested by a lower first-passage attack rate and a slight prolongation of incubation time relative to the ARQ/ARQ derived agent.

## Discussion

The efficient transmissions observed in orally challenged ARR/ARR animals demonstrate that this genotype does not provide substantial resistance against the ovine c-BSE agent. Our results strongly contrast with those previously obtained in ARR/ARR and ARR/ARQ sheep orally challenged with cattle c-BSE, where no clinical signs and no or limited PrP^Sc^ accumulation has been evidenced, whereas positive transmission occurred in ARQ/ARQ sheep (*22*–*24*).

The inoculation doses used in this study (5 g of brain equivalent material) were similar to those used in studies that concluded the absence of cattle c-BSE transmission through the oral route in ARR/ARR sheep. However, in the absence of an endpoint titration establishing the c-BSE titer in our inoculum, the hypothesis that differences in the infectious titer in the inoculum account for, or at least contribute to, the discrepancies observed between studies cannot be ruled out.

In sheep, the age at the time of inoculation does appear to affect the efficacy of c-BSE transmission in orally exposed ARQ/ARQ sheep (*25*). Transmission efficiency is much higher in animals challenged before weaning (<3 weeks) than in animals inoculated after weaning (>3 months). In our study, lambs were orally challenged 24 hours after birth and at the age of 2 weeks, whereas in previous studies, where no c-BSE transmission to ARR/ARR animals was observed, the age at inoculation varied from 3–6 months (*26*,*27*) or 5–8 months (*22*).

Experimental oral exposure early after birth is potentially more relevant to a scenario where maternal lateral transmission (via milk and contact with placenta) would play a central role in the disease transmission, as observed in classical scrapie–infected flocks (*28*,*29*). Experimental oral challenge after weaning is certainly a relevant model to mimic a scenario where sheep would be exposed to the c-BSE agent through the ingestion of contaminated feedstuffs (meat and bone meal), as observed in cattle during the c-BSE epidemics.

The last major difference between our transmission experiment and those reported in previous studies was the use of an ovine-adapted c-BSE rather than cattle c-BSE as inoculum. The apparent higher capacity of ARQ/ARQ sheep-passaged c-BSE (when compared with cattle c-BSE) to cross transmission barriers (transmission to porcine and human PrP-expressing hosts) is a well-documented phenomenon. The use of such ovine-passaged c-BSE as inoculum could, at least partly, explain the efficient transmission of the c-BSE agent to ARR/ARR sheep.

During the past 20 years, a breeding for resistance policy relying on the progressive increase of the ARR allele frequency in sheep has been implemented by certain member states of the European Union (EU). That policy’s original objectives were to reduce the global incidence of TSEs and to prevent c-BSE emergence and spread in sheep populations. The most recent analysis of the small ruminants’ TSE epidemiologic situation in the EU confirmed that the breeding for resistance policy is an efficient means to reduce classical scrapie prevalence in sheep populations (*10*). However, the transmission of the c-BSE agent to ARR/ARR sheep reported in this study suggests that ARR allele selection could have a more limited effect than originally expected on the risk for c-BSE propagation in the sheep population.

At the clinical stage of the disease, the distribution and levels of c-BSE prions in the peripheral tissues of both ARR/ARR and ARQ/ARQ experimentally challenged animals were broadly similar. The main differences observed between both genotypes were a slower dissemination of the c-BSE agent in the organism and a longer incubation period in the ARR/ARR animals.

In the humanized transgenic mouse panel, both ARR/ARR- and ARQ/ARQ-derived c-BSE remained transmissible to mice expressing methionine 129 human PrP^C^, confirming that neither ovine genotype eliminates zoonotic potential. However, the ARR/ARR isolate exhibited modestly reduced transmission efficiency, evident as a lower primary attack rate and longer mean survival times, compared with its ARQ/ARQ counterpart. Those kinetic differences were largely lost after a single adaptation passage, however, suggesting that once the species barrier is crossed, the underlying strain behaves similarly. 

In 2001, specific risk material (SRM) measures were implemented throughout the EU, consisting of the systematic removal of cattle and small ruminants’ tissues susceptible to contain critical levels of prion infectivity from the food and feed chains. The SRM measures are key for ensuring the protection of consumers against exposure to prions present in farmed animals. Current SRM measures applied to small ruminants in the EU consist of the removal of the spleen and the ileum and, in animals over 12 months of age, the skull (including the eyes and brain), spinal cord, and tonsils. Because of the large distribution of TSE infectivity in the lymphoid tissues of small ruminants, SRM measures applied to sheep and goats are considered to have a more limited effect on the protection of consumers than they have in the cattle c-BSE context (*30*). However, mathematical modeling of the effect of the SRM measures on the different prion diseases susceptible to occur in small ruminants (atypical scrapie, classical scrapie, and c-BSE) confirmed the strong positive effect of the SRM measures on the final consumer exposure to these different prions (*31*).

In conclusion, although the capacity of the c-BSE agent to propagate in ARR/ARR sheep can be considered unfortunate news, the continuation of the TSE surveillance and SRM measures currently in force for small ruminants in the EU will continue to ensure efficient protection against the risk for exposure to this zoonotic agent. c-BSE infection in ARR/ARR sheep can still pose a public-health risk, but the quantitative probability of successful cross-species transmission might be lower than transmission associated with ARQ/ARQ sheep cases.

Appendix Additional information about oral transmission of classical bovine spongiform encephalopathy in ARR/ARR sheep.
