# Supplementary material for: Oral Transmission of Classical Bovine Spongiform Encephalopathy in ARR/ARR Sheep
Source: Emerg Infect Dis. 2025 Dec;31(12):2243–53. doi: 10.3201/eid3112.250501 (PMC12782190; doi:10.3201/eid3112.250501)
Supplement: Appendix — Additional information about oral transmission of classical bovine spongiform encephalopathy in ARR/ARR sheep. [file 25-0501-Techapp-s1.pdf]

Article DOI: https://doi.org/10.3201/eid3112.250501
EID cannot ensure accessibility for supplementary materials supplied by authors. Readers who have difficulty accessing supplementary content should contact the authors for assistance.

Oral Transmission of Classical Bovine Spongiform Encephalopathy in ARR/ARR Sheep

Appendix

Appendix Table. End point titration of BSE in sheep reference isolate by bioassay in bovine PrP expressing mice (tgBov) and by Protein Misfolding Cyclic Amplification*

Sheep passaged c-BSE isolate	Bioassay tgBov		PMCA positive reactions (tg ARQ substrate)
	Positive mice	Survival time (days±SD)	
**Neat**	6/6	223 ± 4	NA
**10^{-1}**	6/6	250 ± 9	NA
**10^{-2}**	6/6	290 ± 12	12/12
**10^{-3}**	6/6	338 ± 18	12/12
**10^{-4}**	6/6	386 ± 38	12/12
**10^{-5}**	5/6	486 ± 96	12/12
**10^{-6}**	1/6	402†	12/12
**10^{-7}**	0/6	>700	10/12
**10^{-8}**	0/6	>700	5/12
**10^{-9}**	NA	NA	1/12
**10^{-10}**	NA	NA	0/12
**10^{-11}**	NA	NA	0/12

A 10% (weight/volume) brain homogenate was prepared using the brainstem of a clinically affected ARQ/ARQ genotype sheep inoculated with BSE. Groups of six tgBov mice were intracerebrally inoculated with 20 µL of serial ten-fold dilutions of this homogenate. Mice were considered positive when proteinase K-resistant PrP (PrP^{res}) deposition was detected in the brain by Western blot. Survival times (in days) are presented as mean±SD, except for dilutions marked with an asterisk (*), which indicate cases where less than half of the mice were scored as positive. The same dilution series was used to seed PMCA reactions, with 5 µL per reaction. Twelve replicates were performed for each sample dilution. PMCA substrate was derived from transgenic mice overexpressing the ARQ variant of sheep prion protein (tgARQ/tgShXI). Reactions were subjected to three rounds of amplification. After each round, the reaction products (1 volume) were mixed with fresh substrate (9 volumes) to seed the next round. After the third round, PMCA reaction products were analyzed by Western blot for PrP^{res} detection. The number of PrP^{res}-positive reactions versus the total number of reactions is reported.

*NA, not available

†Dilution with survival time in days

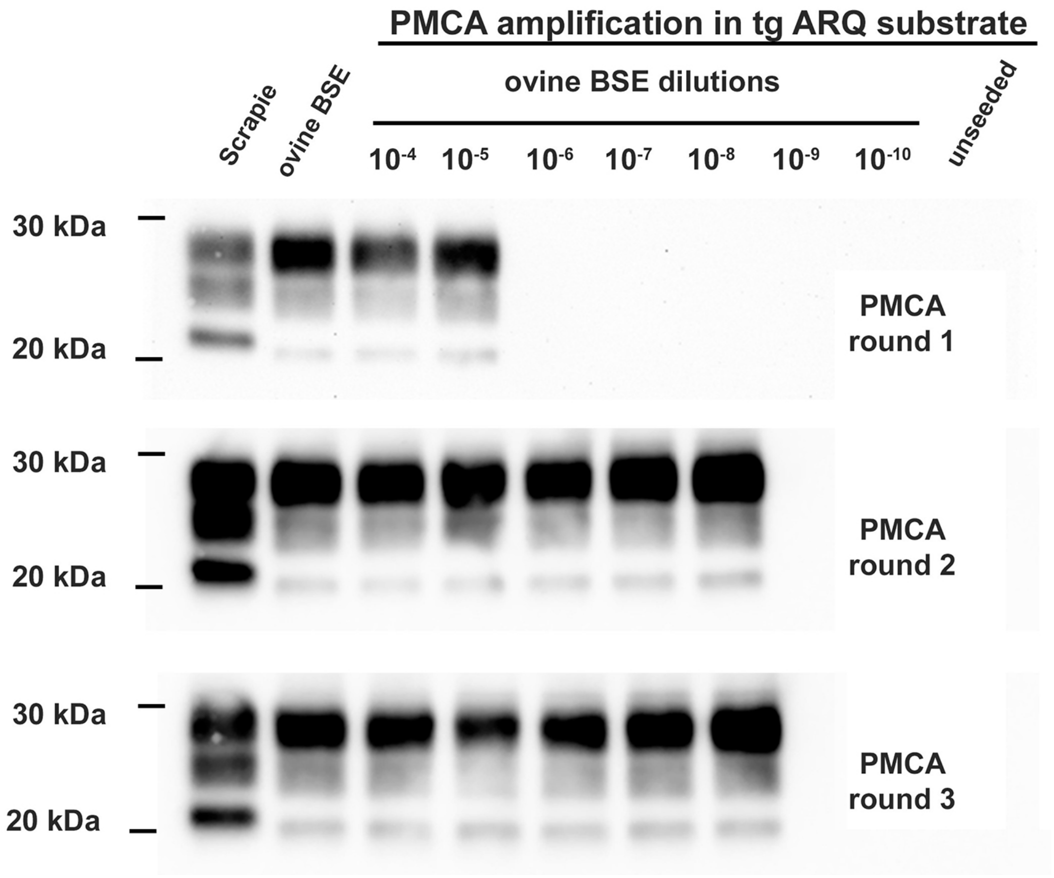

Appendix Figure. PMCA amplification of ovine BSE agent. PMCA reactions were seeded with a dilution series (10% weight / volume -10^{-2} to 10^{-10} dilution) of a reference ovine BSE brain homogenate, previously titrated by bioassay in tgBov mice (intracerebral route – $10^{7.2}$ LD_{50}/mL). PMCA substrate was prepared using brains from transgenic mice overexpressing the ARQ variant of sheep PrP (tgARQ/tgShXI). Unseeded reactions were used as specificity controls. Reactions underwent three to four amplification rounds (96 cycles: 10s sonication followed by 14 min 50s incubation at 39.5°C). After each round, products were split: one part to seed the next round and another for Western blot analysis of PrP^{res} (Sha31 antibody). A sheep scrapie isolate was included as a Western blot control.
